# Manganese superoxide dismutase as a diagnostic marker for malignant pleural mesothelioma

**DOI:** 10.1054/bjoc.1999.1037

**Published:** 2000-02-01

**Authors:** K Kahlos, P Pääkkö, E Kurttila, Y Soini, V L Kinnula

**Affiliations:** Departments of Internal Medicine and Pathology, University of Oulu, and Oulu University Hospital, Kajaanintie 50, Oulu, FIN-90220, Finland

**Keywords:** mesothelioma, metastatic adenocarcinoma, diagnosis, manganese superoxide dismutase, immunohistochemistry

## Abstract

Although several immunohistochemical markers are available, differential diagnosis between mesothelioma and metastatic adenocarcinoma of the pleura is difficult. We have found that the immunoreactivity of manganese superoxide dismutase (MnSOD), an important antioxidant enzyme, is high in mesothelioma compared to healthy pleural mesothelium. The aim of the present study was to investigate whether MnSOD can be used in the differential diagnosis of malignant mesothelioma and metastatic adenocarcinoma of the pleura. MnSOD expression was assessed by using immunohistochemistry in biopsies of malignant mesothelioma (*n* = 35) and metastatic adenocarcinoma of the pleura (*n* = 21). MnSOD immunoreactivity was assessed semiquantitatively with and without microwave pretreatment. Fifteen of the 35 malignant mesotheliomas showed moderate or strong MnSOD expression without and 23 with microwave pretreatment, the corresponding figures for metastatic adenocarcinoma of the pleura being 1 and 2 out of 21 (*P* = 0.002 and *P*< 0.001, respectively by Fisher's exact test). Only mesothelioma biopsies showed strong MnSOD reactivity, and it was never negative in mesothelioma, whereas one-third of the adenocarcinomas showed no MnSOD reactivity. In conclusion, MnSOD immunoreactivity can, combined with other markers, aid the differential diagnosis between malignant mesothelioma and metastatic adenocarcinoma of the pleura. © 2000Cancer Research Campaign


*British Journal of Cancer* (2000) **82**(5), 1022–1029
© 2000 Cancer Research Campaign
DOI: 10.1054/ bjoc.1999.1037, available online at http://www.idealibrary.com on IDEAL®

### Manganese superoxide dismutase as a diagnostic marker for malignant pleural mesothelioma

**K Kahlos[1], P Pääkkö[2], E Kurttila[1], Y Soini[2] and VL Kinnula[1]**

Departments of [1]Internal Medicine and [2]Pathology, University of Oulu, and Oulu University Hospital, Kajaanintie 50, FIN-90220, Oulu, Finland

**Summary** Although several immunohistochemical markers are available, differential diagnosis between mesothelioma and metastatic adenocarcinoma of the pleura is difficult. We have found that the immunoreactivity of manganese superoxide dismutase (MnSOD), an important antioxidant enzyme, is high in mesothelioma compared to healthy pleural mesothelium. The aim of the present study was to investigate whether MnSOD can be used in the differential diagnosis of malignant mesothelioma and metastatic adenocarcinoma of the pleura. MnSOD expression was assessed by using immunohistochemistry in biopsies of malignant mesothelioma ($n$ = 35) and metastatic adenocarcinoma of the pleura ($n$ = 21). MnSOD immunoreactivity was assessed semiquantitatively with and without microwave pretreatment. Fifteen of the 35 malignant mesotheliomas showed moderate or strong MnSOD expression without and 23 with microwave pretreatment, the corresponding figures for metastatic adenocarcinoma of the pleura being 1 and 2 out of 21 ($P$ = 0.002 and $P$ < 0.001, respectively by Fisher's exact test). Only mesothelioma biopsies showed strong MnSOD reactivity, and it was never negative in mesothelioma, whereas one-third of the adenocarcinomas showed no MnSOD reactivity. In conclusion, MnSOD immunoreactivity can, combined with other markers, aid the differential diagnosis between malignant mesothelioma and metastatic adenocarcinoma of the pleura. © 2000 Cancer Research Campaign

**Keywords:** mesothelioma; metastatic adenocarcinoma; diagnosis; manganese superoxide dismutase; immunohistochemistry

Malignant mesothelioma is a tumour that originates from the mesothelial cells of serous cavities, and is mostly associated with occupational exposure to asbestos fibres (Mossman et al, 1996). A typical feature of mesothelioma is its resistance to chemotherapeutic agents and radiation. A number of different tumours, which metastasize in pleura may mimic mesothelioma. The most common problem in this respect is adenocarcinoma. The differential diagnosis between mesothelioma and adenocarcinoma is still difficult.

The diagnosis of mesothelioma is usually based on a typical histopathology combined with several immunohistochemical markers, which are positive in adenocarcinoma but not in mesothelioma. The most common markers are B72.3, CD15 (LeuM1), carcinoembryonal antigen (CEA), BER-Ep4, MOC-31 and epithelial membrane antigen (EMA) (Gaffey et al, 1992; Arber and Weiss, 1993; Demjek and Hjerpe, 1994; Ruitenbeek et al, 1994; Leers et al, 1998). As far as we know, calretinin may be the only histochemical marker that is positive in human mesothelioma and negative in adenocarcinoma (Leers et al, 1998). Another marker which may possibly be used in the differential diagnosis of mesothelioma is manganese superoxide dismutase (MnSOD); we have recently observed that the mRNA and specific activities of MnSOD are highly elevated in mesothelioma cell line cells compared to non-malignant mesothelial cells (Kinnula et al, 1996). Furthermore, a more recent study carried out in our laboratory showed that the MnSOD protein is highly expressed in tumour biopsies of malignant mesothelioma (Kahlos et al, 1998).

MnSOD is a superoxide radical scavenging mitochondrial enzyme, which is crucial in protecting cells and tissues against high oxygen tension and oxidants (Tsan et al, 1990; Wispe et al, 1992). Various cell types of the lung parenchyma have unique antioxidant enzyme profiles (Kinnula et al, 1995). MnSOD reactivity has been detected in type II pneumocytes and alveolar macrophages in healthy human lung (Coursin et al, 1996; Lakari et al, 1998). It is low or undetectable in healthy human pleural mesothelium (Kahlos et al, 1998) and weakly stained in human bronchial epithelium (Kinnula et al, 1994; Coursin et al, 1996; Lakari et al, 1998). MnSOD is induced by cytokines (Wong and Goeddel, 1988; Tsan et al, 1992) and asbestos fibres (Mossman et al, 1986; Janssen et al, 1994) in vitro and by hyperoxia in vivo, especially in rat pleural mesothelium (Clyde et al, 1993). Very little is known about the expression of MnSOD in human lung tumours. Coursin and co-workers showed that the immunoreactivity of MnSOD is variable in human lung adenocarcinoma (Coursin et al, 1996). With the exception of our recent study (Kahlos et al, 1998), there are no previous reports on MnSOD immunoreactivity in pleural tumours.

The aim of this study was to compare the level of MnSOD as determined by immunohistochemistry with semiquantitation in malignant pleural mesothelioma and metastatic adenocarcinoma of the pleura, and to test the hypothesis that MnSOD could be used as an additional marker in the diagnosis of human mesothelioma.

### MATERIALS AND METHODS

### Patients, handling of specimens, and diagnostic aspects

Histopathologically typical cases of malignant mesothelioma and metastatic adenocarcinoma of the pleura were retrieved from the

*Received 6 May 1999*
*Revised 26 August 1999*
*Accepted 26 August 1999*

*Correspondence to:* VL Kinnula files of the Department of Pathology, Oulu University Hospital, by re-evaluating thoracoscopic pleural biopsies obtained during 1976–1997. Fifty-six patients (11 women and 45 men), of whom 35 had a malignant mesothelioma and 21 a metastatic adenocarcinoma of the pleura, were included in the study. Of the 24 mesothelioma patients with adequate data available, 16 (67%) had evidence of occupational exposure to asbestos as established from the patient history. The biopsy material was fixed in 10% formalin and the specimens were then dehydrated and embedded in paraffin.

The malignant mesotheliomas were classified into epithelial, sarcomatoid (fibrous, i.e. spindle-cell) or biphasic subtypes (WHO, 1981). The malignant mesotheliomas were distinguished from the metastatic adenocarcinomas of the pleura on the basis of the presence of intracellular or extracellular hyaluronic acid in mesothelioma, while the adenocarcinomas usually contained intracellular, mostly globular, PAS-positive and diastase-resistant epithelial mucin. In problematic cases diagnostic electron microscopy was performed to demonstrate typical long microvilli in malignant mesotheliomas, but not in metastatic adenocarcinomas (Hammar, 1994). Immunohistochemical staining against certain antigens, such as cytokeratins, EMA and CEA, has been increasingly used in the differential diagnosis between malignant mesothelioma and metastatic adenocarcinoma of the pleura. Accordingly, metastatic adenocarcinomas of the pleura were diagnosed on the basis of CEA-positivity and often contained intracytoplasmic EMA-positivity, while malignant mesotheliomas were CEA-negative and occasionally exhibited membrane-bound positivity for EMA (van der Kwast et al, 1988; Hammar, 1994).

### Cell cultures

Mesothelioma cell lines (M14K, M24K, M25K, M28K, M33K, M38K) have been established from the tumour tissue of six untreated mesothelioma patients and one (M10K) from a metastasis of mesothelioma in a patient who had received both radiation and chemotherapy before sample (Pelin-Enlund et al, 1990; Pelin et al, 1994; Ungar et al, 1994). Human lung adenocarcinoma A549 cells and non-malignant SV40 transformed Met5A (Ke et al, 1989) mesothelial cells were obtained from American Type Culture Collection (Rockville, MD, USA). The mesothelioma and Met5A cells were grown in RPMI-1640 medium supplemented with 10% fetal calf serum, 100 U $ml^{-1}$ penicillin, 100 μg $ml^{-1}$ streptomycin and 0.03% L-glutamine (all from LTI Life Technologies, Paisley, UK) at 37°C in a 5% carbon dioxide atmosphere. The A549 cells were grown in similar atmospheric conditions in minimum essential medium supplemented with L-glutamine and penicillin–streptomycin as above (all from LTI Life Technologies).

### Immunohistochemistry

One representative paraffin block was selected for immunohistochemical stainings. Four-micron-thick sections were cut and processed further within a few days. They were deparaffinized in xylene, and re-hydrated in a descending ethanol series. Endogenous peroxidase was consumed by incubating the sections in 0.1% hydrogen peroxide in methanol for 10 min. In order to enhance the immunoreactivity for MnSOD, the microwave-pretreatment antigen retrieval technique was also applied to each specimen. Before incubation with the primary antibody the slides were placed in a 0.1 M citrate buffer (pH 6.0) and boiled for 2 min at 850 W, and after that for 3 min at 350 W. A polyclonal antibody for MnSOD was a gift from Prof. James D Crapo (National Jewish Medical Center, Denver, CO, USA). This antihuman recombinant MnSOD (rh MnSOD) antibody has been prepared by immunizing rabbits with rh MnSOD (Boehringer Mannheim, Indianapolis, IL, USA). The antiserum in immunoblots produces only one band at a molecular weight of MnSOD, and preincubation of the antiserum with rh MnSOD abolishes the reaction (Kinnula et al, 1994). The sections were incubated with the primary antibody (anti-MnSOD with a dilution 1:1000) at room temperature for 2 h, followed by a biotinylated swine anti-rabbit secondary antibody (at a dilution of 1:200 for 30 min) and the avidin–biotin–peroxidase complex (both from Dakopatts, Glostrup, Denmark). The colour was developed with diaminobenzidine. The sections were counter-stained with a light haematoxylin stain. The negative controls were established substituting phosphate-buffered saline (PBS) at pH 7.2 and normal rabbit serum for the primary antibody.

### Western blotting

The cells were mixed with the electrophoresis sample buffer and boiled for 5 min at 95°C; 50 μg of cell protein was applied per lane to a 12% sodium dodecyl sulphate polyacrylamide gel (Laemmli, 1970). The gel was electrophoresed for 1.5 h (90 V) at room temperature, the protein was transferred (45 min, 100 V) onto Hybond ECL nitrocellulose membranes (Amersham, Arlington Heights, IL, USA) in a Mini-PROTEAN II Cell (Bio-Rad, Hercules, CA, USA). The blotted membrane was incubated with rabbit antibody to recombinant human MnSOD (1:10 000) (Dr JD Crapo) followed by donkey anti-rabbit secondary antibody (1:30 000) conjugated to horseradish peroxidase (Amersham). MnSOD was detected by an enhanced chemiluminescence system (ECL; Amersham), and the luminol excitation was imaged on X-ray film. β-actin expression of the cells was detected by reprobing the same membranes with a monoclonal anti-actin antibody (1:2500) (Sigma, St Louis, MO, USA) followed by sheep anti-mouse antibody conjugated to horseradish peroxidase (1:3000) (Amersham). Cell protein was measured using the Bio-Rad method (Bradford, 1976).

### Light microscopical evaluation

For immunohistochemical staining the whole tissue section was evaluated by light microscopy and the results were assessed semi-quantitatively by grading the staining intensity of the tumour cells as follows: negative (–), weak (+), moderate (++), and strong (+++) immunoreactivity. When microwave antigen retrieval was used, the percentage of the whole cell population showing any immunoreactivity was also evaluated. All the sections were evaluated blindly by two of the authors.

### Statistical analysis

SPSS 7.0 for Windows (Chicago, IL, USA) was used for the statistical analyses. The significance between the groups was compared using Fisher's exact test. The survival of the patients in relation to MnSOD reactivity was assessed by the log-rank and Breslow tests. *P*-values of less than 0.05 were considered statistically significant.

© 2000 Cancer Research Campaign

**Table 1** Clinical information on patients with malignant mesothelioma and MnSOD immunoreactivity of the tumours without and after microwave antigen retrieval

| Patient no. | Histological type | Sex | Age | MnSOD immunoreactivity without microwave retrieval | MnSOD immunoreactivity after microwave retrieval |
|---|---|---|---|---|---|
| 1 | Epithelial | M | 75 | – | +++ |
| 2 | Epithelial | F | 70 | – | ++ |
| 3 | Epithelial | M | 63 | – | ++ |
| 4 | Epithelial | M | 73 | – | + |
| 5 | Epithelial | M | 64 | – | + |
| 6 | Epithelial | M | 54 | – | + |
| 7 | Epithelial | M | 78 | – | + |
| 8 | Epithelial | M | 70 | – | + |
| 9 | Epithelial | M | 63 | + | ++ |
| 10 | Epithelial | M | 59 | + | + |
| 11 | Epithelial | M | 57 | + | ++ |
| 12 | Epithelial | M | 51 | ++ | ++ |
| 13 | Epithelial | M | 46 | ++ | +++ |
| 14 | Epithelial | M | 67 | ++ | ++ |
| 15 | Epithelial | M | 78 | ++ | ++ |
| 16 | Epithelial | M | 68 | ++ | +++ |
| 17 | Epithelial | M | 56 | +++ | +++ |
| 18 | Epithelial | M | 66 | +++ | +++ |
| 19 | Epithelial | M | 50 | +++ | +++ |
| 20 | Epithelial | M | 73 | +++ | +++ |
| 21 | Epithelial | M | 59 | +++ | +++ |
| 22 | Epithelial | M | 79 | +++ | +++ |
| 23 | Biphasic | M | 57 | – | ++ |
| 24 | Biphasic | F | 57 | ++ | +++ |
| 25 | Biphasic | M | 70 | ++ | +++ |
| 26 | Biphasic | M | 68 | ++ | + |
| 27 | Sarcomatoid | F | 42 | – | + |
| 28 | Sarcomatoid | M | 59 | – | + |
| 29 | Sarcomatoid | M | 63 | – | ++ |
| 30 | Sarcomatoid | M | 52 | – | + |
| 31 | Sarcomatoid | F | 33 | – | ++ |
| 32 | Sarcomatoid | M | 71 | – | ++ |
| 33 | Sarcomatoid | M | 67 | – | ++ |
| 34 | Sarcomatoid | F | 55 | + | + |
| 35 | Sarcomatoid | M | 73 | ++ | + |

### RESULTS

The MnSOD immunoreactivity values and clinical characteristics of the patients with pleural malignant mesothelioma or metastatic adenocarcinoma are shown in Tables 1 and 2. Fifteen of the 35 malignant mesotheliomas without microwave pretreatment and 23 after microwave antigen retrieval showed moderate or strong MnSOD immunoreactivity (Table 1). The figures for metastatic adenocarcinoma of the pleura were one and two out of 21 respectively (Table 2). Thus, malignant mesotheliomas showed significantly more often moderate or strong MnSOD immunoreactivity than metastatic adenocarcinomas of the pleura either without ($P$ = 0.002 by Fisher's exact probability test; Table 3) or after microwave antigen retrieval ($P$ < 0.001 by Fisher's exact probability test; Table 4). The histological type of the mesothelioma also appeared to affect MnSOD immunoreactivity; strong MnSOD immunoreactivity was observed only in epithelial and biphasic but not in sarcomatoid mesotheliomas ($P$ = 0.03 by Fisher's exact test without microwave pretreatment, $P$ = 0.02 by Fisher's exact test after microwave antigen retrieval; Table 1). Some MnSOD reactivity could also be detected in the stromal cells of mesothelioma (not shown) and adenocarcinomas (Figure 1 C,D). Even though microwave antigen retrieval clearly improved MnSOD immunoreactivity, it did not change the characteristic staining pattern of the tumours (Figure 1). The improvement of MnSOD immunoreactivity obtained by microwave antigen retrieval in both tumour types and especially in malignant mesotheliomas is shown in Tables 5 and 6. The reactive mesothelium, which was seen in a few cases along with tumour tissue, also showed prominent immunoreactivity for MnSOD. In these cases immunoreactivity in the surrounding reactive mesothelium was clearly stronger than in the metastatic adenocarcinomas (Figure 2). MnSOD reactivity did not differ between the mesothelioma patients with or without previous exposure to asbestos fibres. No staining was seen in the negative controls, where the primary antibody had been replaced by rabbit serum or PBS.

The immunoreactivity of MnSOD in mesothelioma was also assessed in relation to the survival of the patients. The short-term, but not the long-term, survival of the mesothelioma patients with moderate or strong MnSOD immunoreactivity after microwave retrieval was slightly better than those with negative or weak MnSOD immunoreactivity (median survival 8 and 2 months respectively, $P$ = 0.057 by log-rank test, $P$ = 0.008 by Breslow test, Figure 3). No significant difference in survival was seen between the patients with epithelial, biphasic or sarcomatoid subtypes of mesothelioma ($P$ = 0.18 by log-rank test).

 *© 2000 Cancer Research Campaign*

**Table 2** Clinical information on patients with metastatic adenocarcinoma of the pleura and MnSOD immunoreactivity in tumours without and after microwave antigen retrieval

| Patient no. | Primary tumour | Sex | Age | MnSOD immunoreactivity without microwave | MnSOD immunoreactivity after microwave retrieval |
|---|---|---|---|---|---|
| 1 | Bile duct | M | 65 | + | + |
| 2 | Breast | F | 43 | – | + |
| 3 | Breast | F | 65 | – | + |
| 4 | Breast | F | 70 | + | + |
| 5 | Kidney | F | 66 | – | + |
| 6 | Kidney | M | 53 | + | – |
| 7 | Lung | M | 68 | – | – |
| 8 | Lung | F | 60 | – | – |
| 9 | Lung | M | 55 | – | + |
| 10 | Lung | M | 63 | – | – |
| 11 | Lung | M | 49 | – | + |
| 12 | Lung | M | 69 | – | + |
| 13 | Lung | M | 68 | – | – |
| 14 | Lung | M | 76 | – | + |
| 15 | Lung | M | 71 | – | – |
| 16 | Lung | M | 64 | – | + |
| 17 | Lung | M | 60 | + | + |
| 18 | Lung | M | 49 | + | + |
| 19 | Lung | M | 79 | + | – |
| 20 | Lung | F | 65 | ++ | ++ |
| 21 | Unknown | M | 75 | + | ++ |

**Table 3** MnSOD immunoreactivity of malignant mesotheliomas and metastatic adenocarcinomas of the pleura without microwave pretreatment

| Tumour type | MnSOD immunoreactivity | |
|---|---|---|
| | Negative or Weak (– or +) No. of cases | Moderate or Strong (++ or +++) No. of cases |
| Mesothelioma | 20 | 15 |
| Metastatic adenocarcinoma of the pleura | 20 | 1 |

$P = 0.002$ by Fisher's exact test.

The expression of MnSOD was also investigated in non-malignant and malignant cell lines, including non-malignant human pleural mesothelial (Met5A) cells, lung adenocarcinoma A549 cells and seven pleural mesothelioma cell lines. Western blotting revealed low MnSOD expression in mesothelial cells and adenocarcinoma cells, while the immunoreactivity of MnSOD was prominent, albeit variable, in all malignant mesothelioma cell line cells (Figure 4). The antibody showed excellent detection of the MnSOD protein with no background staining or impurities.

### DISCUSSION

These results indicate that MnSOD immunoreactivity may be used as an additional diagnostic marker in the differential diagnosis between malignant mesothelioma and metastatic adenocarcinoma of the pleura. All mesotheliomas showed at least some MnSOD immunoreactivity (when a microwave pretreatment technique was used), while metastatic adenocarcinomas of the pleura tended to be either negative or only slightly positive. MnSOD immunoreactivity was strong only in mesotheliomas but not in any of the metastatic adenocarcinomas investigated.

Numerous studies have indicated that MnSOD is usually low in malignant tumours, whereas the expression of other antioxidant enzymes is variable (Oberley and Buettner, 1979; Oberley and Oberley, 1997). In addition to pleural mesothelioma (Kahlos et al, 1998) certain tumours of the central nervous system, thyroid gland, kidney and gastrointestinal tract also contain high levels of MnSOD compared to their non-malignant counterparts (Nishida et al, 1993; Cobbs et al, 1996; Oberley and Oberley, 1997; Janssen et al, 1998). In some malignancies MnSOD expression appears to be associated with the degree of differentiation of the tumour (Landriscina et al, 1996). To our knowledge there is only one study in which MnSOD immunoreactivity has been examined in human lung adenocarcinoma. In that particular study the three biopsies of adenocarcinoma showed variable MnSOD immunoreactivities, and in agreement with our results both the tumour and stromal cells were positive for MnSOD (Coursin et al, 1996). So far, no biopsies of mesothelioma have been included in that or any other study, with the exception of our recent study on human mesothelioma (Kahlos et al, 1998). A previous study on rat lung showed that the mRNA of MnSOD is highly up-regulated, especially in the mesothelium of hyperoxia-exposed rats (Clyde et al, 1993). In agreement with this, we found that MnSOD immunoreactivity was enhanced in non-malignant reactive mesothelium, but not in non-malignant airway epithelium. The prominent induction of MnSOD, especially in reactive mesothelial cells, is unclear but

© 2000 Cancer Research Campaign 

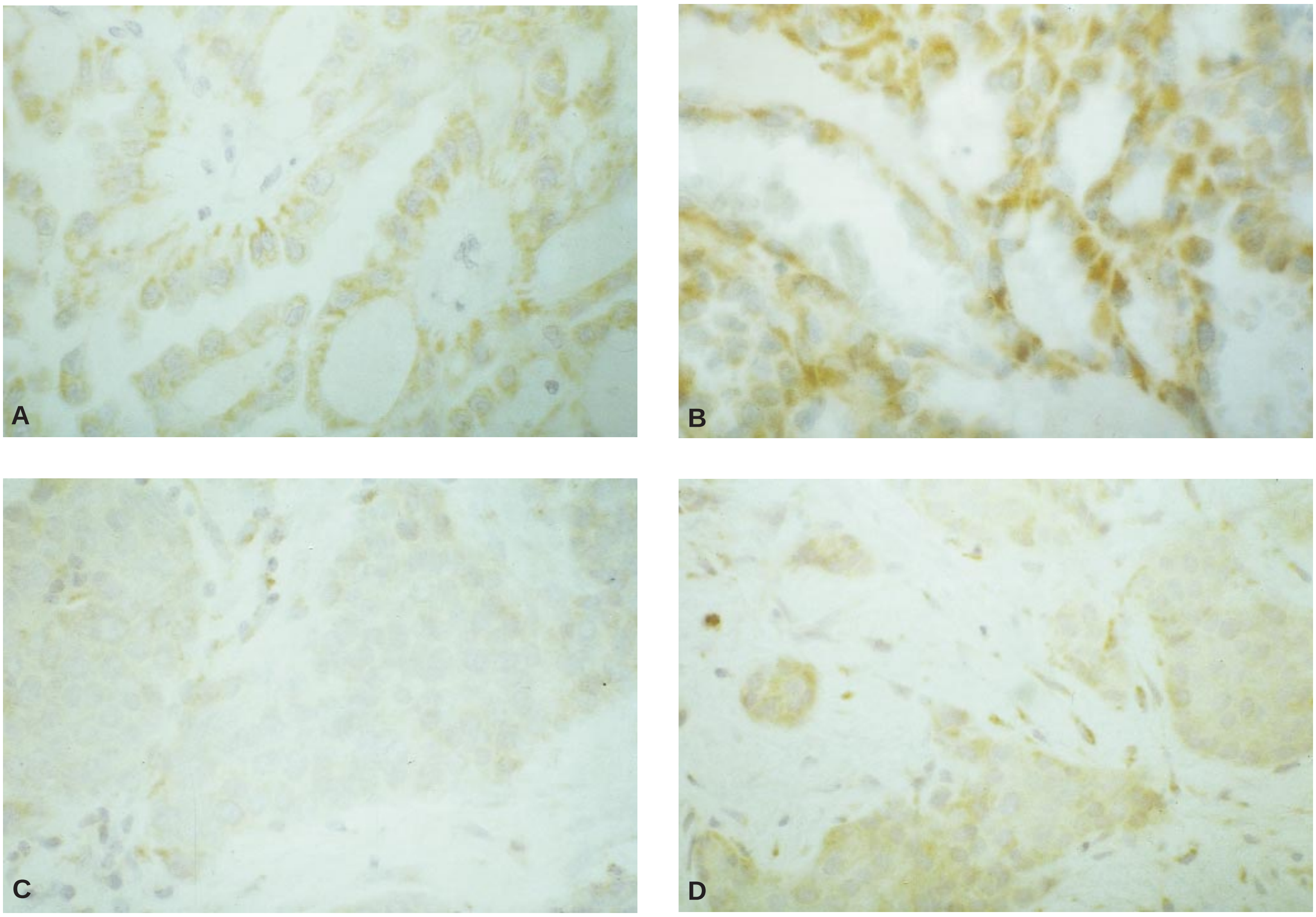

**Figure 1** MnSOD immunoreactivity in epithelial malignant mesothelioma (**A, B**) and metastatic adenocarcinoma of the pleura (**C, D**) without (**A, C**) and after microwave antigen retrieval (**B, D**). The MnSOD immunostaining is clearly stronger after microwave pretreatment. Magnification × 400

**Table 4** MnSOD immunoreactivity of mesotheliomas and metastatic adenocarcinomas of the pleura after microwave antigen retrieval

| Tumour type | MnSOD immunoreactivity | |
|---|---|---|
| | Negative or Weak (– or +) No. of cases | Moderate or Strong (++ or +++) No. of cases |
| Mesothelioma | 12 | 23 |
| Metastatic adenocarcinoma of pleura | 19 | 2 |

$P < 0.001$ by Fisher's exact test.

**Table 5** MnSOD immunoreactivity of mesotheliomas without and after microwave antigen retrieval

| | MnSOD immunoreactivity | | |
|---|---|---|---|
| | Negative (–) (No. of cases) | Weak (+) (No. of cases) | Moderate or strong (++ or +++) (No. of cases) |
| Without microwave pretreatment | 16 | 4 | 15 |
| After microwave antigen retrieval | 0 | 12 | 23 |

Negative vs weak-strong: $P < 0.00001$ by Fisher's exact test, negative-weak vs moderate-strong: $P = 0.046$ by Fisher's exact test.

*© 2000 Cancer Research Campaign*

**Table 6** MnSOD immunoreactivity of metastatic adenocarcinoma of the pleura without and after microwave antigen retrieval

| | MnSOD immunoreactivity | | |
|---|---|---|---|
| | **Negative (–) (No. of cases)** | **Weak (+) (No. of cases)** | **Moderate or strong (++ or +++) (No. of cases)** |
| Without microwave pretreatment | 13 | 7 | 1 |
| After microwave antigen retrieval | 6 | 13 | 2 |

Negative vs weak-strong: $P = 0.03$ by Fisher's exact test, negative-weak vs moderate-strong: $P = 0.5$ by Fisher's exact test.

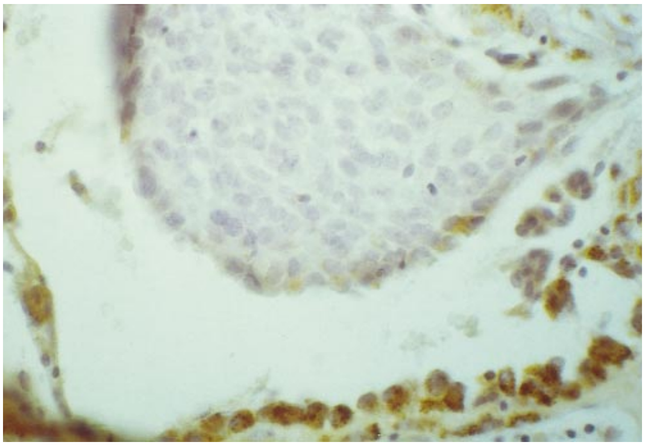

**Figure 2** MnSOD immunoreactivity in reactive mesothelium in comparison with metastatic adenocarcinoma of the pleura. Reactive mesothelium below shows stronger immunoreactivity for MnSOD than the metastatic adenocarcinoma above. Magnification ×400

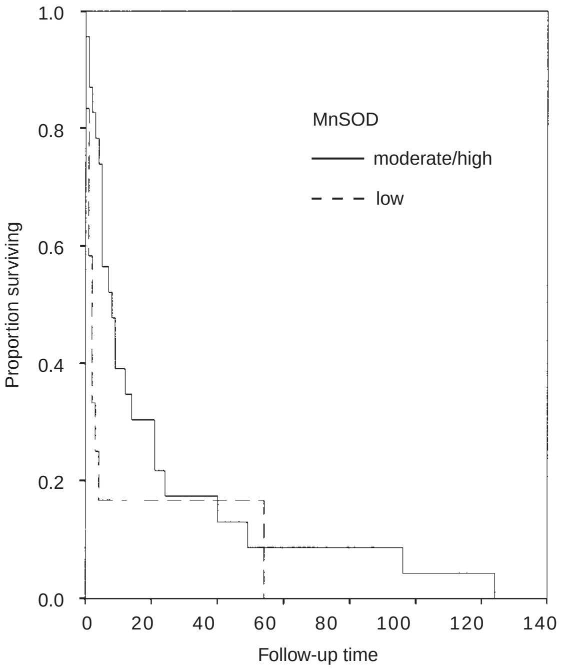

**Figure 3** Survival in relation to MnSOD immunoreactivity in malignant mesothelioma. The patients with malignant mesothelioma showing moderate or strong MnSOD immunoreactivity after microwave antigen retrieval had a tendency for better short term prognosis than those with negative or weak MnSOD immunoreactivity ($P = 0.057$ by log-rank test, $P = 0.006$ by Breslow test)

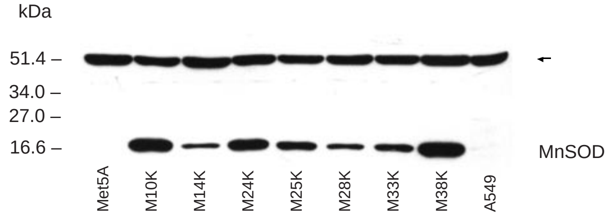

**Figure 4** A representative Western blot analysis showing moderate or high MnSOD immunoreactive protein (21 kDa) in seven mesothelioma cell lines (M10K, M14K, M24K, M25K, M28K, M33K, M38K), and low MnSOD immunoreactivity in non-malignant mesothelial Met5A cells and in lung adenocarcinoma A549 cells. β-actin expression was detected to confirm the loading homogeneity (arrow)

consistent with previous studies showing that MnSOD is highly up-regulated by cytokines such as tumour necrosis factor-α (TNF-α) (Wong and Goeddel, 1988; Kinnula et al, 1995) also in Met5A cells (Pietarinen-Runtti et al, 1996). Since MnSOD may be induced both in reactive and malignant mesothelium, it cannot be used in the differential diagnosis between non-malignant reactive mesothelium and malignant mesothelioma.

Not only the type of the tumour, but also the immunohistochemical technique itself may have effects on the intensity of MnSOD immunoreactivity. Non-specific immunoreactivity had been excluded by reactions with rabbit serum and PBS. Since histochemical methods are semiquantitative and may vary in different laboratories, all samples were examined blindly by two observers both with and without microwave pretreatment for antigen retrieval. Van Driel and co-workers reported low MnSOD expression in colorectal neoplasms, and in that study no microwave pretreatment was used (Van Driel et al, 1997). In the study of Cobbs and co-workers malignant nervous system tumours showed prominent MnSOD immunoreactivity without microwave pretreatment (Cobbs et al, 1996). In the study of Coursin and co-workers no such pretreatment was used, and the immunoreactivities for MnSOD in their three adenocarcinoma biopsies were variable (Coursin et al, 1996). In our recent study no such treatment was used either and MnSOD was highly stained in all the six mesothelioma biopsies and negative in normal mesothelium (Kahlos et al, 1998). In the present study a remarkable portion of mesothelioma biopsies were negative without microwave antigen retrieval. The staining was intensified with the microwave pretreatment, so that none of the mesothelioma biopsies remained negative, whereas one-third of the 21 metastatic adenocarcinoma biopsies still remained negative. Furthermore, one-third of the mesothelioma biopsies showed strong MnSOD immunoreactivity,

© 2000 Cancer Research Campaign 

while MnSOD immunoreactivity was never strong in adenocarcinomas. Thus strong MnSOD immunoreactivity supports the diagnosis of mesothelioma. If the MnSOD immunoreactivity is weak or negative, the differential diagnosis between mesothelioma and adenocarcinoma remains unclear.

Mesothelioma is mostly associated with exposure to asbestos fibres, and asbestos fibres are known to induce MnSOD in a variety of cells (Mossman et al, 1986; Clyde et al, 1993). The history of previous asbestos exposure was not associated with the intensity of MnSOD immunostaining. However, retrospective information on the exposure of mesothelioma patients to asbestos may underestimate the number of exposed patients. In our previous study, MnSOD reactivity in mesothelioma cells was not associated with the fibre content of the lung (Kahlos et al, 1998). Furthermore, mesothelioma can also develop on patients who have not been exposed to asbestos fibres and even in lungs with low or undetectable levels of asbestos fibres.

Given that antioxidant enzymes, such as superoxide dismutase, protect cells against oxidant stress, they may also have prognostic significance in malignant diseases. Recently, Van Driel and co-workers did not find differences in the survival of their patients with colorectal carcinoma in relation to CuZnSOD immunoreactivity (Van Driel et al, 1997). On the other hand, Janssen and co-workers suggested that high MnSOD reactivity in gastrointestinal cancer may be associated with poor survival (Janssen et al, 1998). The present study suggested that high MnSOD immunoreactivity might be associated with a better short-term prognosis. However, the differences in survival were very small. Furthermore, the prognosis of mesothelioma patients is very poor as was also observed in this study.

Even though the reactivities of various antioxidant enzymes are known to be decreased in cultured cells (Kinnula et al, 1992, 1994), this is not the case in mesothelioma cell line cells. We have previously found that several mesothelioma cell line cells have higher mRNA level and specific activity of MnSOD than non-malignant Met5A mesothelial cells or whole-lung homogenate (Kinnula et al, 1996). In agreement with these findings, this study showed that all the seven mesothelioma cell lines had higher MnSOD reactivities than Met5A cells. This result is also consistent with our recent finding showing very low reactivity in Met5A cells, weak reactivity in M14K cells and strong reactivity in M38K cells immunocytochemically when this same antibody has been used (Kahlos et al, 1998). A549 cells which have originally been established from alveolar epithelial type II pnemocytes also contained lower MnSOD immunoreactivity than any of the investigated mesothelioma cells.

In conclusion, high MnSOD is characteristic of human malignant mesothelioma, and MnSOD immunohistochemistry can aid the differential diagnosis of mesothelioma and metastatic adenocarcinoma.

### ACKNOWLEDGEMENTS

We thank Prof JD Crapo for providing the antibody to human MnSOD, and Dr Kaija Linnainmaa from the Occupational Health Institute of Helsinki for providing the mesothelioma cell line cells. The skilful technical assistance of Mr Manu Tuovinen and Mrs Raija Sirviö are gratefully acknowledged. This work was partly supported by the Finnish Anti-Tuberculosis Association Foundation and the Sigrid Juselius Foundation.

### REFERENCES

Arber DA and Weiss LM (1993) CD15: A review. *Appl Immunohistochem* **1**: 17–30

Bradford M (1976) A rapid and sensitive method for the quantitation of microgram quantities of protein utilizing the principal of protein-dye binding. *Anal Biochem* **72**: 248–254

Clyde BL, Kang BH, Slot JW, Vincent R and Crapo JD (1993) Distribution of manganese superoxide dismutase mRNA in normal and hyperoxic rat lung. *Am J Respir Cell Mol Biol* **8**: 530–537

Cobbs CS, Levi DS, Aldape K and Israel MA (1996) Manganese superoxide dismutase expression in human central nervous system tumors. *Cancer Res* **56**: 3192–3195

Coursin DB, Chilia HP, Sempf J, Oberley TD and Oberley LW (1996) An immunohistochemical analysis of antioxidant and glutathione S-transferase enzyme levels in normal and neoplastic human lung. *Histol Histopathol* **11**: 851–860

Demjek A and Hjerpe A (1994) Carcinoembryonic antigen-like reactivity in malignant mesothelioma. *Cancer* **73**: 464–469

Gaffey MJ, Mills SE, Swanson, PE, Zarbo RJ, Shah AR and Wick MR (1992) Immunoreactivity for BER-EP4 in adenocarcinomas, adenomatoid tumours and malignant mesotheliomas. *Am J Surg Pathol* **16**: 593–599

Hammar SP (1994) Pleural diseases. In: *Pulmonary Pathology*, Dail DA and Hammar SP (eds), pp. 1463–1579. Springer-Verlag: New York

Janssen AML, Bosman CB, Sier CFM, Griffioen G, Kubben FJGM, Lamers CBHW, van Krieken JHJM, van de Velde CJH and Verspaget HW (1998) Superoxide dismutases in relation to the overall survival of colorectal cancer patients. *Br J Cancer* **78**: 1051–1057

Janssen YMW, Marsh JP, Driscoll KE, Borm PJA, Odersdörster G and Mossman BT (1994) Increased expression of manganese-containing superoxide dismutase in rat lungs after inhalation of inflammatory and fibrogenic minerals. *Free Rad Biol Med* **16**: 315–322

Kahlos K, Anttila S, Asikainen T, Kinnula K, Raivio KO, Mattson K, Linnainmaa K and Kinnula VL (1998) Manganese superoxide dismutase in healthy pleural mesothelium and in malignant pleural mesothelioma. *Am J Respir Cell Mol Biol* **18**: 570–580

Ke Y, Reddel RR, Gerwin BI, Reddel HK, Somers ANA, McMenamin MG, LaVeck MA, Stahel RA, Lechner JF and Harris CC (1989) Establishment of a human in vitro mesothelial cell model system for investigating mechanisms of asbestos-induced mesothelioma. *Am J Pathol* **134**: 979–991

Kinnula VL, Chang L-Y, Everitt JI and Crapo JD (1992) Oxidants and antioxidants in alveolar epithelial type II cells: in situ, freshly isolated, and cultured cells. *Am J Physiol* **262**: L69–L77

Kinnula VL, Yankaskas JR, Chang LY, Virtanen I, Linnala A, Kang BH and Crapo JD (1994) Primary and immortalized (BEAS2B) human bronchial epithelial cells have significant antioxidative capacity in vitro. *Am J Respir Cell Mol Biol* **11**: 568–576

Kinnula VL, Crapo JD and Raivio KO (1995) Generation and disposal of reactive oxygen metabolites in the lung. *Lab Invest* **73**: 3–19

Kinnula VL, Pietarinen-Runtti P, Kahlos K, Pelin K, Mattson K and Linnainmaa K (1996) Manganese superoxide dismutase in human pleural mesothelioma cell lines. *Free Rad Biol Med* **21**: 527–532

Laemmli VK (1970) Cleavage of structural proteins during the assembly of the head bacteriophage T4. *Nature* **227**: 680–684

Lakari E, Pääkkö P and Kinnula VL (1998) Manganese superoxide dismutase, but not CuZn superoxide dismutase, is highly expressed in the granulomas of pulmonary sarcoidosis and extrinsic allergic alveolitis. *Am J Respir Crit Care Med* **158**: 589–596

Landriscina M, Remiddi, F, Ria F, Palazzotti B, de Leo ME, Iacoangeli M, Rosseli R, Scerrati M and Galeotti T (1996) The level of MnSOD is directly correlated with grade of brain tumours of neuroepithelial origin. *Br J Cancer* **74**: 1877–1885

Leers MPG, Aarts MMJ and Theunissen PHMH (1998) E-cadherin and calretin: a useful combination of immunohistochemical markers for differentiation between mesothelioma and metastatic adenocarcinoma. *Histopathology* **32**: 209–216

Mossman BT, Marsh JP and Shatos MA (1986) Alteration of superoxide dismutase activity in tracheal epithelial cells by asbestos and inhibition of cytotoxicity by antioxidants. *Lab Invest* **54**: 204–212

Mossman BT, Kamp DW and Weitzman SA (1996) Mechanisms of carcinogenesis and clinical features of asbestos-associated cancers. *Cancer Invest* **14**: 466–480

Nishida S, Akai F, Iwasaki H, Hosokawa K, Kusunoki T, Suzuki K, Taniguchi N, Hashimoto S and Tamura TT (1993) Manganese superoxide dismutase content and localization in human thyroid tumors. *J Pathol* **169**: 341–345

*© 2000 Cancer Research Campaign*

Oberley LW and Buettner GR (1979) Role of superoxide dismutase in cancer: a review. *Cancer Res* **39**: 1141–1149
Oberley LW and Oberley TD (1997) Role of antioxidant enzymes in the cancer phenotype. In: *Lung Biology in Health and Disease*, Clerch LB and Massaro DJ (eds) vol. 105, pp. 279–308. Marcel Dekker: New York
Pelin K, Hirvonen A and Linnainmaa K (1994) Expression of cell adhesion molecules and connexins in gap junctional intercellular communication competent primary mesothelial cells. *Carcinogenesis* **15**: 2673–2675
Pelin-Enlund K, Husgafvel-Pursiainen K, Tammilehto L, Klockars M, Jantunen K, Gerwin BI, Harris CC, Tuomi T, Vanhala E, Mattson K and Linnainmaa K (1990) Asbestos-related malignant mesothelioma: growth, cytology, tumorigenicity, and consistent chromosome findings in cell lines from five patients. *Carcinogenesis* **11**: 673–681
Pietarinen-Runtti P, Raivio KO; Linnainmaa K, Ekman A, Saksela M and Kinnula VL (1996) Differential effects of tumor necrosis factor and asbestos fibers on manganese superoxide dismutase induction and oxidant-induced cytotoxicity in human mesothelial cells. *Cell Biol Toxicol* **12**: 167–175
Ruitenbeek T, Gouw ASH and Poppema S (1994) Immunocytology of body cavity fluids: MOC-31, a monoclonal antibody discriminating between mesothelial and epithelial cells. *Arch Pathol Lab Med* **118**: 265–269
Tsan MF, White JE, Santana TA and Lee CY (1990) Tracheal insufflation of tumor necrosis factor protects rats against oxygen toxicity. *J Appl Physiol* **68**: 1211–1219
Ungar S, van de Meeren A, Tammilehto L, Linnainmaa K, Mattson K and Gerwin BI (1994) High levels of MDM2 are not correlated with the presence of wild-type p53 in human malignant mesothelioma cell lines. *Br J Cancer* **74**: 1534–1540
van der Kwast TH, Versnel MA, Delahaye M, de Jong A, Zondervan PE and Hoogsteden H (1988) Expression of epithelial membrane antigen on malignant mesothelioma cells. An immunocytochemical and immunoelectron microscopic study. *Acta Cytol* **32**: 169–174
van Driel BEM, Lyon H, Hoogenraad DCJ, Anten S, Hansen U and van Noorden CJF (1997) Expression of CuZn- and Mn-superoxide dismutase in human colorectal neoplasms. *Free Rad Biol Med* **23**: 435–444
Wispe JR, Warner BB, Clark JC, Dey CR, Neuman S, Glasser S, Crapo JD, Chang L-Y and Whitsett JA (1992) Human Mn-superoxide dismutase in pulmonary epithelial cells of transgenic mice confers protection from oxygen injury. *J Biol Chem* **267**: 23937–23941
Wong GH and Goeddel DV (1988) Induction of manganese superoxide dismutase by tumor necrosis factor: possible protective mechanism. *Science* **242**: 941–944
WHO (1981) *Histological Typing of Lung Tumours. International Classification of Tumours*, 2nd edn. World Health Organization: Geneva

*© 2000 Cancer Research Campaign*
